# Supplementary material for: Comparison of Grip Strength in Recreational Climbers and Non-Climbing Athletes—A Cross-Sectional Study
Source: Int J Environ Res Public Health. 2020 Dec 27;18(1):129. doi: 10.3390/ijerph18010129 (PMC7796164; doi:10.3390/ijerph18010129)
Supplement: Supplementary file 1 [file ijerph-18-00129-s001.pdf]

## Supplemental Materials

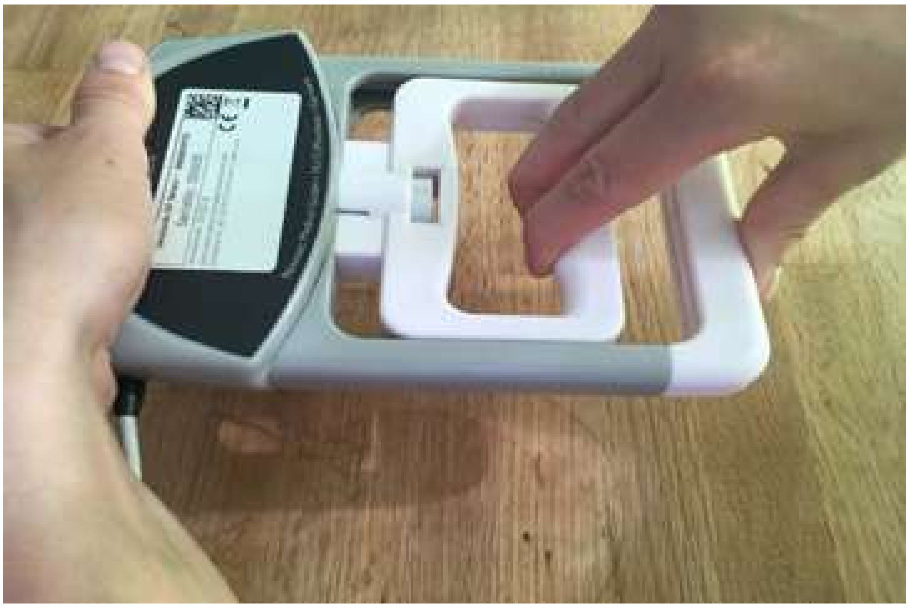

**Supplementary Figure S1. The Leonardo mechanograph® GF (Novotec Medical GmbH, Pforzheim, Germany) was used for grip force measurements.** An example of measurement in a sitting position with 90° flexed elbow and pronated hand is demonstrated with a Pinch I/II + III of the dominant hand.

**Supplementary Table S1.** Demographics for the climbing and non-climbing group.

| | Climbers (n=50) | Non-Climbers (n=50) | p |
|---|---|---|---|
| Age (yrs) | 30.26 [26.7-33.9] | 26.42 [23.8-29.0] | n.s. (0.1008)[1] |
| Sex | 68% male, 32% female | 62% male, 38% female | n.s. (0.6753)[2] |
| Height (m) | 176.0 [173.5-178.6] | 178.4 [175.7-181.0] | n.s. (0.3177)[1] |
| Weight (kg) | 67.0 [63.0-70.9] | 73.2 [69.6-76.8] | n.s. (0.0581)[1] |
| BMI ($kg/m^2$) | 22.0 [21.3-22.6] | 22.9 [22.2-23.6] | n.s. (0.0575)[1] |
| Ape index | 1.01 [1.0-1.1] | 1.01 [1.0-1.0] | n.s. (0.3580)[3] |
| Climbing experience (yrs) | 6.80 [4.7-8.9] | 0[0-0] | - |
| Training frequency (per week) | 2.5 [2.2-2.9] | 2.4 [1.9-3.0] | n.s. (0.1332)[1] |

[1]Mann-Whitney test, [2]Fisher's exact text, [3]Unpaired t test.

**Supplementary Table S2.** Analysis of pinch grips between climbers and non-climbers adapted to BMI. Both groups are compared (unpaired t-test) and effect size is reported (ES).

| | Climbers | Non-Climbers | | |
|---|---|---|---|---|
| | Dom | | p-value | ES |
| Pinch I/II (kg) | 0.3984 [0.36-0.43] | 0.34 [0.31-0.37] | *** (0.0003) | moderate |
| Pinch I/III (kg) | 0.4149 [0.38-0.45] | 0.3477 [0.32-0.38] | ** (0.0024) | moderate |
| Pinch I/IV (kg) | 0.3075 [0.27-0.34] | 0.2423 [0.22-0.27] | ** (0.011) | moderate |
| Pinch I/III+IV (kg) | 0.5257 [0.48-0.57] | 0.4381 [0.40-0.47] | ** (0.0024) | moderate |
| Pinch I/II+III (kg) | 0.6062 [0.56-0.65] | 0.5108 [0.47-0.55] | ** (0.0037) | moderate |
| Pinch I/II+III+IV (kg) | 0.645 [0.59-0.70] | 0.5387 [0.50-0.58] | *** (0.0004) | moderate |
| Fist (kg) | 1.981 [1.82-2.14] | 1.792 [1.66-1.92] | * (0.0116) | small |
| | **Climbers** | **Non-Climbers** | | |
| | **Non-dom** | | **p-value** | **ES** |
| Pinch I/II (kg) | 0.3635 [0.33-0.40] | 0.2959 [0.27-0.32] | ** (0.0026) | moderate |
| Pinch I/III (kg) | 0.3808 [0.34-0.42] | 0.3087 [0.28-0.34] | ** (0.0091) | moderate |
| Pinch I/IV (kg) | 0.2885 [0.26-0.32] | 0.2248 [0.21-0.24] | ** (0.0025) | moderate |
| Pinch I/III+IV (kg) | 0.4909 [0.45-0.54] | 0.4015 [0.37-0.44] | ** (0.0040) | moderate |
| Pinch I/II+III (kg) | 0.5438 [0.50-0.59] | 0.4681 [0.43-0.50] | ** (0.0025) | moderate |
| Pinch I/II+III+IV (kg) | 0.6219 [0.57-0.68] | 0.5006 [0.47-0.54] | *** (0.0006) | moderate |
| Fist (kg) | 1.881 [1.72-2.04] | 1.625 [1.50-1.75] | n.s. (0.0724) | moderate |

***p<0.001, **p<0.01, *p<0.05.
